# A Comparative Study on the Minimal Invasiveness of Full-Endoscopic and Microendoscopic Cervical Foraminotomy Using Intraoperative Motor Evoked Potential Monitoring

**DOI:** 10.3390/medicina56110605

**Published:** 2020-11-11

**Authors:** Masahiro Hirahata, Tomoaki Kitagawa, Muneyoshi Fujita, Ryutaro Shiboi, Hirotaka Kawano, Hiroki Iwai, Hirohiko Inanami, Hisashi Koga

**Affiliations:** 1 Department of Orthopaedic Surgery, Teikyo University School of Medicine, 2-11-1 Kaga, Itabashi-ku, Tokyo 173-8605, Japan; pleasure-masa@cyber.ocn.ne.jp (M.H.); muneyoshi.fujita.0302@main.teikyo-u.ac.jp (M.F.); hkawano-tky@umin.net (H.K.); 2 Department of Orthopaedic Surgery, Oono Central Hospital, 3-20-3 Shimokaizuka, Ichikawa-shi, Chiba 272-0821, Japan; stubtender@yahoo.co.jp; 3 Department of Neurosurgery, Iwai FESS Clinic, 8-18-4 Minamikoiwa, Edogawa-ku, Tokyo 133-0056, Japan; rocky5477@gmail.com (H.I.); hkoga0808@gmail.com (H.K.); 4 Department of Orthopaedic Surgery, Iwai Orthopaedic Medical Hospital, 8-17-2 Minamikoiwa, Edogawa-ku, Tokyo 133-0056, Japan; inanamihiro@gmail.com; 5 Department of Orthopaedic Surgery, Inanami Spine and Joint Hospital, 3-17-5 Higashishinagawa, Shinagawa-ku, Tokyo 140-0002, Japan

**Keywords:** intraoperative motor evoked potential monitoring, full-endoscopic cervical foraminotomy, microendoscopic cervical foraminotomy, cervical radiculopathy

## Abstract

*Background and Objectives:* Full-endoscopic cervical foraminotomy (FECF) and microendoscopic cervical foraminotomy (MECF) are effective surgeries for cervical radiculopathy and are considered minimally invasive in terms of damage to paraspinal soft tissue. However, no studies have quantitatively compared FECF and MECF in terms of neurological invasiveness. The aim of this study was to compare the neurological invasiveness of FECF and MECF using intraoperative motor evoked potential (MEP) monitoring. *Materials and Methods:* A chart review was conducted of 224 patients with cervical radiculopathy who underwent FECF or MECF between April 2014 and March 2020. Patients were 37 women and 187 men, with a mean age of 51 (range, 21–86) years. FECF was performed in 143 cases and MECF was performed in 81 cases. *Results:* Average MEP amplitude significantly increased from 292 mV before to 677 mV after nerve root decompression in patients who underwent the FECF. The average improvement rate was 273%. In patients who underwent the MECF, average MEP amplitude significantly increased from 306 mV before to 432 mV after nerve root decompression. The average improvement rate was 130%. The improvement rate was significantly higher for FECF compared with MECF. *Conclusions:* MEP amplitude increased after nerve root decompression in both FECF and MECF, but the improvement rate was higher in FECF. These results suggest that FECF might be more minimally invasive than MECF in terms of neurological aspects.

## 1. Introduction

Cervical radiculopathy is caused by compression of the nerve root at the cervical foramen. This condition typically presents as arm pain and can cause sensory and motor deficits along the path of innervation of the affected nerve root [1]. Conservative treatment should be attempted for all patients with new-onset radiculopathy in the absence of a significant motor deficit, but patients with persistent symptoms require surgical treatment [2].

Cervical foraminotomy, which was first described in 1945 by Spurling and Scoville, is regarded as an effective surgery for cervical radiculopathy. With the flourishing of theories and techniques that require minimal invasiveness in spine surgery, Adamson reported microendoscopic cervical foraminotomy (MECF) in 2001 [3] and Ruetten et al. reported full-endoscopic cervical foraminotomy (FECF) in 2007. FECF is less invasive than MECF and is attracting growing attention in the development of minimally invasive techniques [4,5,6]. No significant differences between these two approaches has been noted in pooled outcomes for clinical success rate, complication rate, and reoperation rate [7]. However, no comparative study has quantitatively evaluated the minimal invasiveness of FECF and MECF in terms of neurological function. Recent studies have shown that nerve decompression results in an increase in intraoperative transcranial electrical stimulation motor evoked potential (MEP) amplitude [8,9]. The aim of this study was to compare the neurological invasiveness of FECF and MECF using intraoperative MEP monitoring.

## 2. Materials and Methods

### 2.1. Patients

A chart review was conducted of 224 patients with cervical radiculopathy who underwent FECF or MECF between April 2014 and March 2020. A single surgeon performed all FECFs and decided the preoperative and postoperative care. Several expert surgeons performed MECFs and decided the preoperative and postoperative care. Indications for surgery were (1) arm pain caused by nerve root compression lasting ≥6 weeks, (2) concordance between clinical symptoms and radiological findings of nerve root compression with magnetic resonance imaging (MRI), and (3) failure of nonsurgical treatments. Patients who underwent decompression at a single level were included. Exclusion criteria were (1) myelopathy, (2) unreliable MEP, and (3) lack of follow-up evaluation of arm pain. All patients provided written informed consent after receiving an explanation of the study. The study was conducted in accordance with the Declaration of Helsinki, and the protocol was approved by Teikyo University Ethical Review Board for Medical and Health Research Involving Human Subjects (No: 19-138, approved date: 12 September 2019).

### 2.2. Operative Technique

The operation was performed under general anesthesia (total intravenous anesthesia) and radiographic control with the patient prone. Anesthesia was induced with propofol (3–4 μg/mL), fentanyl (2 μg/kg), and vecuronium (0.12–0.16 mg/kg) and maintained with propofol (3 μg/mL), fentanyl (1 μg/kg/h), and vecuronium (0–0.04 mg/kg/h).

**Figure 1:**
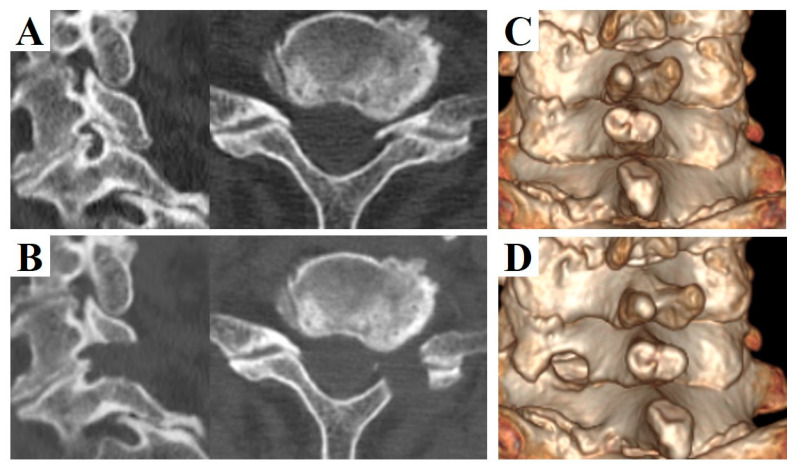
Pre- and postoperative computed tomography (CT) images of a 73-year-old man who underwent left C6/7 full-endoscopic cervical foraminotomy (FECF). (**A**) Preoperative sagittal (left) and axial (right) CT images. (**B**) Postoperative sagittal (left) and axial (right) CT images. (**C**) Pre- and (**D**) postoperative 3-dimensional CT images. Note that the dorsal part of the vertebral foramen was removed after FECF.

FECF: the surgical techniques have been described in detail in our previous reports [10,11]. An 8 mm longitudinal skin incision was made approximately 15 mm lateral to the midline of the operated level. A working sheath with an outer diameter of 7 mm was placed on the cervical lamina after splitting the paravertebral muscles. Under full endoscopic assistance with continuous irrigation, the caudal side of the inferior process of the upper vertebra and the cranial side of the superior process were resected using a surgical drill (Figure 1). Subsequently, the whole circumference of nerve root was carefully exposed. Skin closure was performed without a drainage tube.

MECF: surgical techniques were performed according to Adamson’s report [3]. An 18 mm oblique skin incision was made approximately 20 mm lateral to the midline of the operated level. A sheath with an outer diameter of 16 mm was placed on the cervical lamina after splitting the paravertebral muscles. Under microendoscopic assistance, the caudal side of the inferior process of the upper vertebra and the cranial side of the superior process were resected using a surgical drill. Subsequently, the whole circumference of the nerve root was carefully exposed. Skin closure was performed with a drainage tube.

The patients were permitted to walk 1 day after surgery without a cervical orthosis.

### 2.3. Intraoperative MEP Monitoring

MEP monitoring was performed using the NVM5^TM^ nerve monitoring system (NuVasive INC, San Diego, CA, USA). The transcranial stimulation conditions were as follows: 4–6 train stimuli; stimulus interval, 2 ms; 600–800 mA. The screw-type stimulator was placed 2 cm anterior and 4 cm lateral to Cz (International 10–20 system) over the cerebral cortex motor area. MEPs were recorded from the peripheral limbs via disk electrodes. MEP amplitudes were measured as peak-to-peak voltages. Depending on the operated level, the stimulated muscles were selected from the deltoid (C5), biceps (C6), triceps (C7), and abductor digiti minimi (C8). The improvement value was defined as the difference between the MEP amplitude before decompression and that after decompression. The improvement rate was defined as the improvement value divided by the MEP amplitude before decompression.

### 2.4. Evaluation

The MEP amplitude after nerve root decompression was compared with that before decompression in FECF and MECF. The improvement rate was compared between FECF and MECF. The main outcome of this study was postoperative arm pain rated on the Numerical Rating Scale (NRS). Patients were divided into a satisfactory group (NRS 0–3) and an unsatisfactory group (NRS 4–10) based on guidelines of the National Comprehensive Cancer Network. The cut-off value of the improvement rate was determined using receiver operating characteristic (ROC) curve analysis.

### 2.5. Statistical Analysis

The t-test, Fisher’s exact test, and ROC curve analysis were performed using SAS 9.4 software for Windows (SAS Institute INC, Cary, NC, USA). Statistical significance was set at a *p*-value of < 0.05.

## 3. Results

Patient demographics and clinical characteristics are shown in Table 1. There were 37 women and 187 men, with a mean age of 51 years (range, 21–86). There were 153 patients with cervical spondylotic radiculopathy, 69 with cervical disc herniation, and two with cervical ossification of the posterior longitudinal ligament. FECF was performed in 143 cases, and MECF was performed in 81 cases.

**Figure 2:**
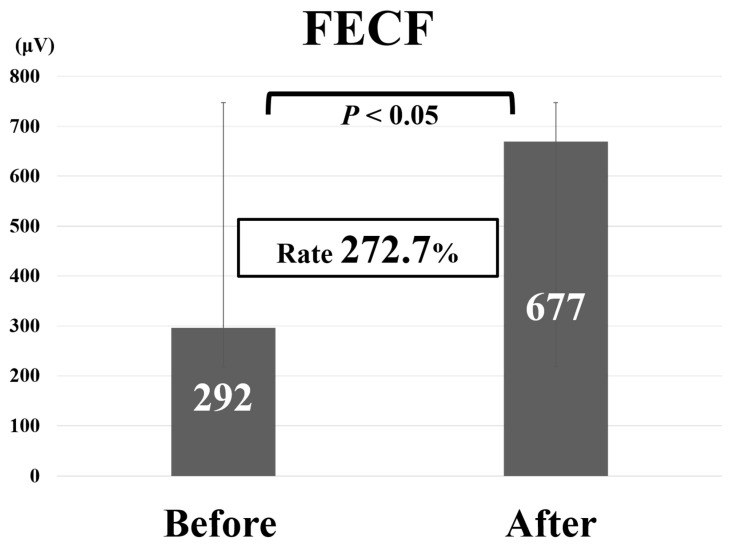
Average motor evoked potential amplitude before and after decompression and average improvement rate in patients who underwent full-endoscopic cervical foraminotomy (FECF).

**Figure 3:**
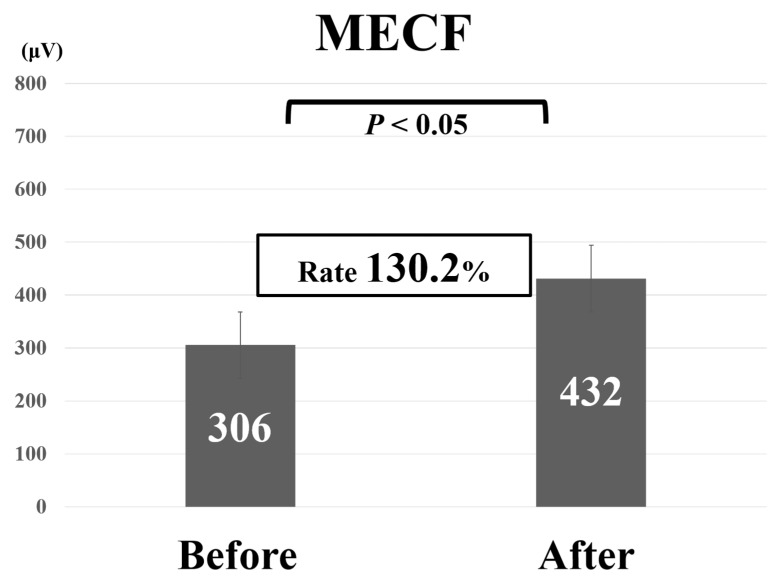
Average motor evoked potential amplitude before and after decompression and average improvement rate in patients who underwent microendoscopic cervical foraminotomy (MECF).

**Figure 4:**
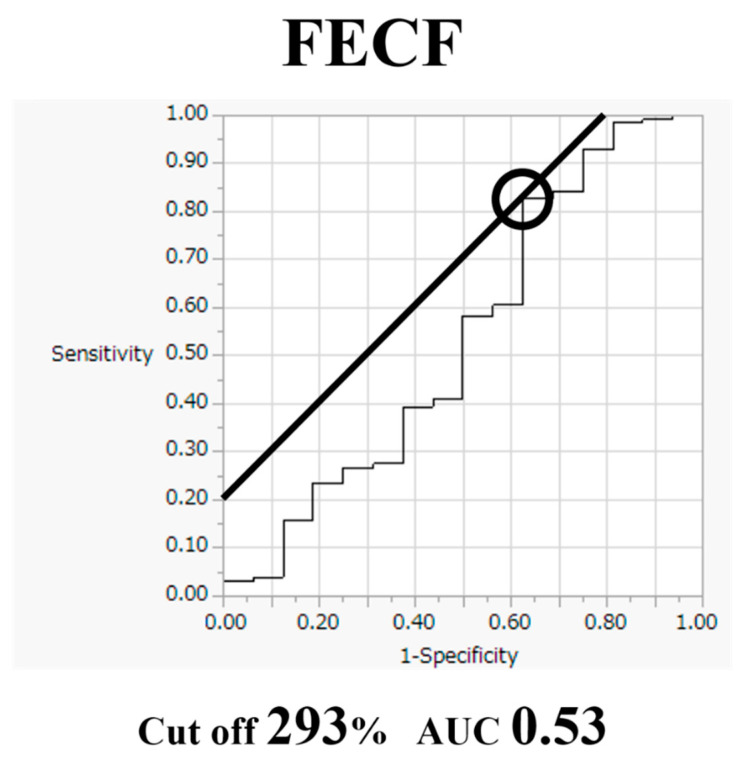
Receiver operating characteristic curve for improvement rate in full-endoscopic cervical foraminotomy (FECF).

**Figure 5:**
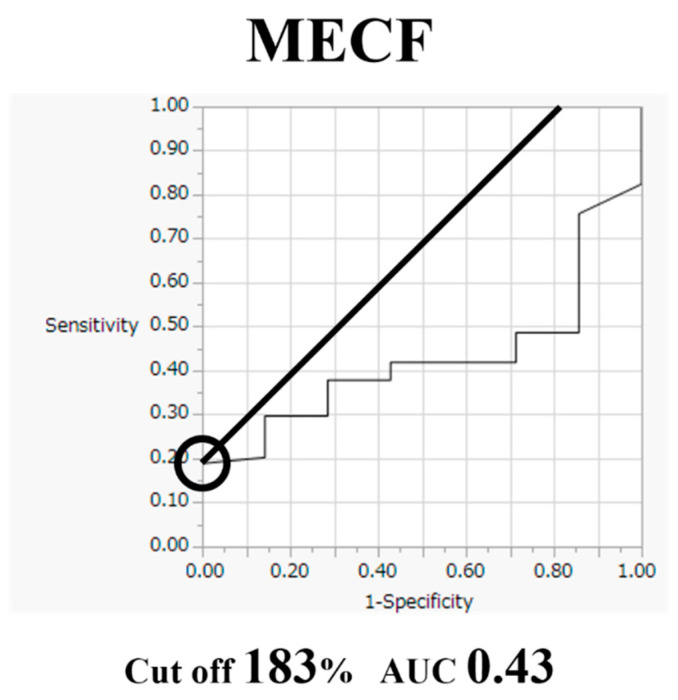
Receiver operating characteristic curve for improvement rate in microendoscopic cervical foraminotomy (MECF).

Comparative findings of FECF and MECF are shown in Table 2. Mean operative time for FECF was 59.4 min (range, 33–104 min). After surgery, arm pain due to disc herniation recurred in one patient. There were no other complications. Mean operative time for MECF was 68.2 min (range, 28–162 min). No patients had complications after MECF. No patients experienced muscle weakness after FECF or MECF. Average MEP amplitude significantly increased from 292 mV before to 677 mV after decompression in patients who underwent FECF. The average improvement rate was 273% (Figure 2). Average MEP amplitude increased from 306 mV before to 432 mV after decompression in patients who underwent the MECF. The average improvement rate was 130.2% (Figure 3). The improvement rate was significantly higher in patients who underwent FECF than in those who underwent MECF. Postoperative pain was satisfactory in 127 patients (88.8%) and unsatisfactory in 16 patients (11.2%) following FECF. Following MECF, postoperative pain was satisfactory in 74 patients (91.4%) and unsatisfactory in seven patients (8.6%). There was no significant difference between FECF and MECF. ROC curve analysis showed that the cut-off value for the improvement rate in FECF was 293% (sensitivity 84%, specificity 33%), with an area under the curve of 0.53 for improvement of NRS arm pain after surgery (Figure 4). ROC curve analysis showed that the cut-off value for the improvement rate in MECF was 183% (sensitivity 19%, specificity 100%), with an area under the curve of 0.43 for improvement of NRS arm pain after surgery (Figure 5).

## 4. Discussion

This study demonstrates that the increase in MEP amplitude after nerve root decompression was greater after FECF than after MECF. Currently, there is a consensus on the need for routine intraoperative MEP monitoring in spine surgery. The American Clinical Neurophysiology Society recommends that surgeons and other members of the operating team should be alerted to the increased risk of severe adverse neurologic outcomes in patients with a decrease in MEP amplitude [12]. The aim of this recommendation is to improve the safety of spine surgery by monitoring for decreases in MEP amplitude, but few studies have focused on increases in MEP amplitude after nerve decompression [13,14]. There are a few reports that MEP amplitude significantly increased after spinal cord decompression. [8,9,15]. In these studies, MEP amplitude was related to clinical outcome measures. However, there are no reports showing a relationship between surgical decompression of the cervical nerve root and intraoperative MEP monitoring. The results of this study can serve as a useful reference to assist physicians in the decision-making regarding surgical decompression of the cervical nerve root. However, this study did not seem to be related to any clinical outcome measures as we found an insignificant difference in the two groups. Therefore, MEP amplitude remained to be clinically significant when it decreased, but not necessarily when it increased.

We found that the improvement rate of FECF was significantly higher than that of MECF. Given that a previous meta-analysis showed that both FECF and MECF are effective and relatively safe treatments for cervical radiculopathy due to lateral disc herniation or osseous foraminal stenosis [7], we expected that this would also be true for nerve root decompression. Accordingly, the difference in MEP improvement rate might be caused by the effect on the nerve root. However, it was difficult to identify why the improvement rate of FECF was higher than that of MECF because there were too many factors that could affect the MEP amplitude. One possible explanation is that the retraction of the nerve root was less traumatic in FECF than in MECF because of the smaller instruments used for FECF. The outer diameter of the microendoscopic working sheath is 16–20 mm, whereas that of the full-endoscopic working sheath is 6–8 mm. Additionally, the imaging medium is air in the microendoscopic optic system but water (normal saline) in the full-endoscopic system, and so the nerve root might be less susceptible to heat and vibration generated by the surgical drill in FECF.

The background of patients who underwent FECF were similar to that of patients who underwent MECF. Pajewski et al. reported that inhalation anesthetics were a factor in decreasing MEP amplitude [16]. In this study, both MECF and FECF were performed under total intravenous anesthesia. Schwartz et al. reported that MEP was extremely sensitive to altered spinal cord blood flow [17]. Causes of decreased spinal cord blood flow include old age, anemia, hypothermia, hypotonia, and hypoxemia. In our previous report on FECF, the estimated blood loss was 0 to 10 mg in all cases, which might be less than that of MECF. Kim et al. reported obesity and increased operative time as factors in decreasing MEP amplitude. In the present study, the mean operative time of FECF was significantly shorter than that of MECF, possibly contributing to the larger increase in MEP amplitude in FECF.

**Figure 6:**
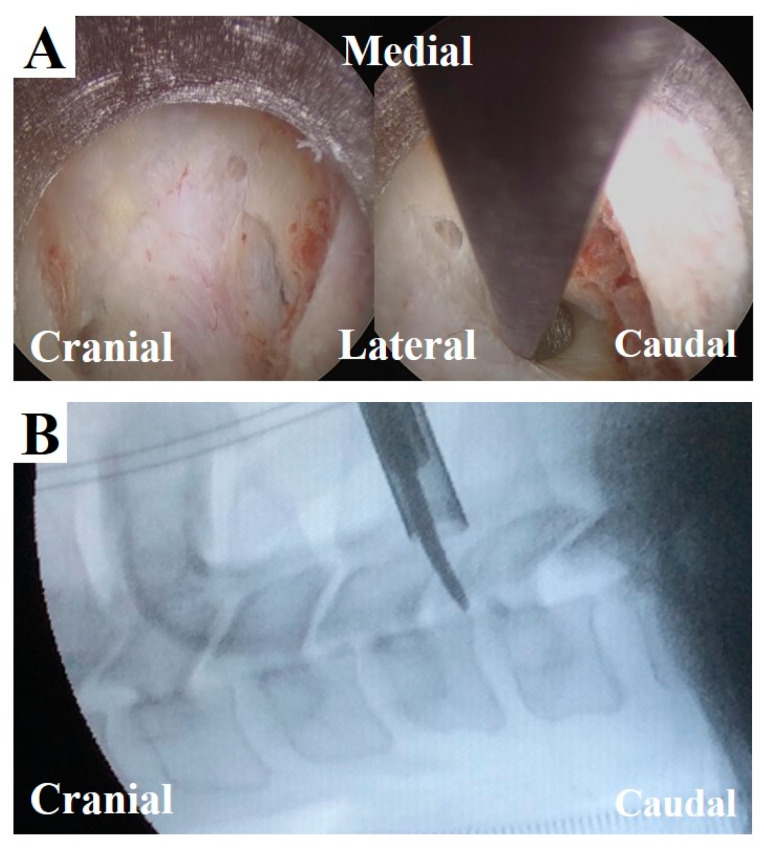
Intraoperative findings and fluoroscopic image at the final stage of FECF. (**A**) After removal of the dorsal part of vertebral foramen, the underlying nerve root could be seen (left). A curved dissector was inserted into the caudal area of the nerve root (right). (**B**) The position of the dissector is confirmed by a fluoroscopic image (lateral view, corresponding to the image in A, right).

The results of this study suggest new criteria for physicians to confirm tension of the nerve root in FECF. Currently, the extent of decompression is confirmed on a lateral fluoroscopic image using a curved dissector (width: 3 mm). When the dissector is inserted into the decompressed foramen, its tip must be located beyond the dorsal border of the vertebral body (Figure 6); otherwise, further decompression is obtained. Although this procedure precisely confirms decompression, it is challenging to limit radiation exposure. From this perspective, intraoperative MEP monitoring might be a good alternative for confirming decompression without radiation exposure.

The cut-off value for the intraoperative MEP improvement rate in FECF was 293%, and that of MECF was 183%. Most previous reports have focused on neurological deterioration, using a 50–80% decrease in MEP amplitude as a criterion [18,19,20,21]. In a prospective multicenter study by the Spinal Cord Monitoring Working Group of the Japanese Society for Spine Surgery and Related Research, Kobayashi et al. reported 3 years’ experience of intraoperative MEP monitoring in 959 spine surgeries. In the study, the alarm point was set at a 70% decrease in amplitude and provided high sensitivity (95%) and specificity (91%) [22]. On the other hand, only two previous studies have focused on neurological improvement or surgical decompression of the spinal cord. In those studies, intraoperative MEP amplitude significantly improved during surgical decompression, and Wang et al. found a cut-off value of 150%, and Wi et al. found a cut-off value of 200% [8,9]. Our result is similar to those, but further data are needed to determine the cut-off point.

Our study has several limitations. First, the only outcome was NRS for arm pain. An additional limitation was that this study was carried out at a single institution. Due to the retrospective non-randomized nature of the study, a multicenter, prospective randomized study is required to confirm the usefulness of intraoperative MEP monitoring in FECF.

## 5. Conclusions

MEP amplitude increased after nerve root decompression in both FECF and MECF, but the improvement rate was higher in FECF. These results suggest that FECF might be more minimally invasive than MECF in terms of neurological aspects.

## Figures and Tables

**Table 1:** Patient demographics and clinical characteristics.

	(*n* = 224)	Percentage
Age, years	51 (21–86)	
Sex		
Female	37	16.5
Male	187	83.5
Diagnosis		
CSR	153	68.3
CDH	69	30.8
OPLL	2	0.9
Surgical procedure		
FECF	143	63.8
MECF	81	36.2
Operated side		
Right	97	43.3
Left	127	56.7
Operated level		
C4/C5	18	8.0
C5/C6	93	41.5
C6/C7	102	45.5
C7/T1	11	4.9

Age is shown as the mean (range); other values are numbers and percentages. CDH, cervical disc herniation; CSR, cervical spondylotic radiculopathy; FECF, full-endoscopic cervical foraminotomy; MECF, microendoscopic cervical foraminotomy; OPLL, ossification of the posterior longitudinal ligament.

**Table 2:** Comparative findings of FECF and microendoscopic cervical foraminotomy (MECF).

	FECF; *n* = 143	MECF; *n* = 81	*p*-Value
Age	51.1 ± 10.1	50.7 ± 11.8	0.82
Female sex	28 (19.6)	9 (11.1)	0.13
Operative time	59.4 ± 15.5	68.2 ± 26.3	0.00
Improvement rate of MEP	272.7 ± 748.1	130.2 ± 377.1	0.03
NRS before surgery	4.7 ± 2.8	4.5 ± 2.8	0.62
NRS after surgery	1.2 ± 1.8	0.8 ± 1.6	0.04

Values are shown as the mean ± standard deviation or number (percent). FECF, full-endoscopic cervical foraminotomy; MECF, microendoscopic cervical foraminotomy; MEP, motor evoked potential; NRS, Numerical Rating Scale.
